# The efficacy of traditional acupuncture on patients with chronic neck pain: study protocol of a randomized controlled trial

**DOI:** 10.1186/s13063-017-2009-1

**Published:** 2017-07-10

**Authors:** Yiling Yang, Xiaoxia Yan, Hongmei Deng, Dian Zeng, Jianpeng Huang, Wenbin Fu, Nenggui Xu, Jianhua Liu

**Affiliations:** 1 Guangzhou University of Traditional Chinese Medicine, 12 Jichang Road, Guangzhou, 510006 People’s Republic of China; 2 The Secondary Medical College, Guangzhou University of Traditional Chinese Medicine, 111 Dade Road, Guangzhou, 510120 People’s Republic of China

**Keywords:** Acupuncture, Chronic pain, Placebo, Sham acupuncture

## Abstract

**Background:**

A large number of randomized trials on the use of acupuncture to treat chronic pain have been conducted. However, there is considerable controversy regarding the effectiveness of acupuncture. We designed a randomized trial involving patients with chronic neck pain (CNP) to investigate whether acupuncture is more effective than a placebo in treating CNP.

**Methods/design:**

A five-arm, parallel, single-blinded, randomized, sham-controlled trial was designed. Patients with CNP of more than 3 months’ duration are being recruited from Guangdong Provincial Hospital of Chinese Medicine (China). Following examination, 175 patients will be randomized into one of five groups (35 patients in each group) as follows: a traditional acupuncture group (group A), a shallow-puncture group (group B), a non-acupoint acupuncture group (group C), a non-acupoint shallow-puncture group (group D) and a sham-puncture group (group E). The interventions will last for 20 min and will be carried out twice a week for 5 weeks. The primary outcome will be evaluated by changes in the Northwick Park Neck Pain Questionnaire (NPQ). Secondary outcomes will be measured by the pain threshold, the Short Form McGill Pain Questionnaire-2 (SF-MPQ-2), the 36-Item Short-Form Health Survey (SF-36) and diary entries. Analysis of the data will be performed at baseline, at the end of the intervention and at 3 months’ follow-up. The safety of acupuncture will be evaluated at each treatment period.

**Discussion:**

The purpose of this trial is to determine whether traditional acupuncture is more effective for chronic pain relief than sham acupuncture in adults with CNP, and to determine which type of sham acupuncture is the optimal control for clinical trials.

**Trial registration:**

Chinese Clinical Trial Registry: ChiCTR-IOR-15006886. Registered on 2 July 2015.

**Electronic supplementary material:**

The online version of this article (doi:10.1186/s13063-017-2009-1) contains supplementary material, which is available to authorized users.

## Background

Chronic neck pain (CNP) is a serious public health and socioeconomic problem worldwide. Studies have shown that the overall prevalence of CNP varies from 0.4 to 86.8% (mean 23.1%) [1]. The prevalence of CNP is greater among office workers than manual workers. This condition is characterized by activity limitations, dizziness, anxiety and insomnia [2]. Physical exercise, massage or physiotherapy, as well as education, have been proved to be efficient in reducing neck pain [3–6]. Acupuncture has also been well accepted as a non-pharmacological treatment for neck pain [7]. However, the treatment effect of acupuncture is moderate [8]. One argument is that “acupuncture is not associated with clinical effects beyond a powerful placebo response” [9, 10].

A growing body of research indicates that clinical trials of acupuncture have encountered many methodological challenges, including the identification of appropriate treatment and control groups, deficiencies in the blinding and follow-up of chronic pain [11]. Firstly, variable acupoints at different sites are commonly selected for the same disease in clinical practice. Based on our clinical experience, more than 20 acupoints have been selected as the main acupoints in neck pain. An appropriate repeatable treatment has not been established. Secondly, although shallow needle insertion and non-acupoints have been widely used in acupuncture research in sham groups, it has been suggested that these methods have therapeutic effects and are adjusted to accommodate different patients according to traditional Chinese medicine (TCM) theory [12]. The academic literature also indicates that the human body has more than 2200 singular points within formal naming beyond meridians [12]. The notion of a non-acupoint is obscure and there may be a role for specific factors of non-acupoints lateral to the acupoints [13]. Moreover, recent academic literature has shown that shallow puncture also produces analgesic effects in pain disorders. Thirdly, it has been reported that about 40% of subjects in these trials could distinguish non-penetrating sham acupuncture from real acupuncture [14].

In view of these doubts, the influence of different needling points and depths should be investigated. First, the acupoints of local lesions including *Jingbailao* (EX-HN 15) and *Jianzhongshu* (SI 15) are commonly used in our hospital for neck pain [15]. The analgesic mechanism of acupoints on local lesions has been established in the spinal segments control theory to explain the gate control theory [16, 17]. According to the innervations of the pain focus, the effects of stimulation of EX-HN 15 and SI 15 on neck analgesia will be studied for feasibility and repeatability [15]. Second, several different groups of non-acupoints and shallow punctures will be established to determine whether traditional acupuncture is more effective for chronic pain relief than sham acupuncture in adults with CNP. The different depth and placement of needle insertion will also be investigated to determine efficacy [18]. Third, a single-blinded practice will be used and the adequacy of subject blinding will also be carefully assessed. The patients will be advised to lie in the prone position on the treatment couch and will be unaware of the treatment course in this trial. Data collection will be completed by the researchers over a 3-month follow-up period. Finally, we will assess the pain threshold (PT) and pain diary as semi-objective outcomes to avoid subjective results.

In this research, a randomized clinical trial will be performed to determine whether the outcome of traditional acupuncture treatment is significantly different to sham acupuncture in relieving CNP. We also aim to investigate which type of sham acupuncture is the optimal control for clinical trials.

## Methods/design

### Study design

**Fig. 1:**
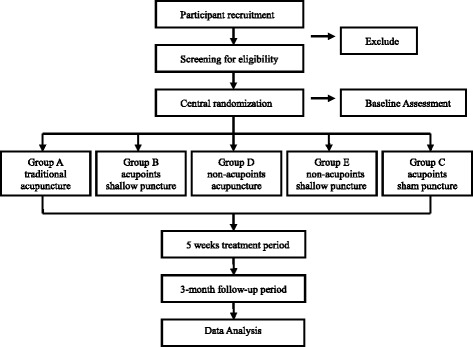
Flowchart of the study design. A total of 175 participants will be randomized to the five groups. The interventions will last for 20 min and will be carried out twice a week for 5 weeks. The study period will be consisted of the baseline, 5 weeks of treatment, and 3-month follow-up

**Fig. 2:**
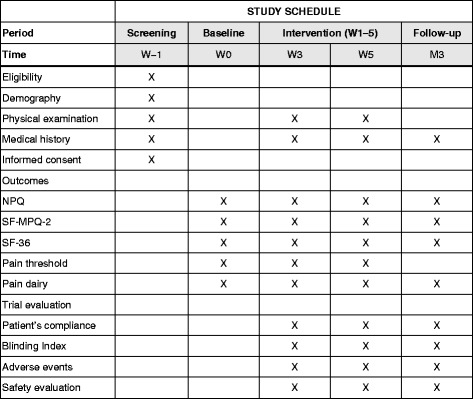
Study schedule. Study protocols on enrollment and time points for assessments using the Standard Protocol Items: Recommendations for Interventional Trials (SPIRIT) figure. W3: the 5th treatment during the 3rd week, W5: the 10th treatment during the 5th week

A single-center randomized controlled trial (RCT) has been designed to compare the differences between traditional acupuncture and sham acupuncture in patients with CNP. In total, 175 patients with CNP will be enrolled. According to the study plan, patients will receive ten treatments over 5 weeks. This trial will be conducted in Guangdong Provincial Hospital of Chinese Medicine from August 2015 to December 2017. The study design is shown in the flowchart in Fig. 1, and the study schedule is presented in Fig. 2.

### Recruitment

The participants will be recruited by bulletin board advertisements in the Traditional Chinese Medicine Hospital of Guangdong Province. Recruitment staff in the acupuncture clinic will be responsible for the enrollment of participants. If a patient with CNP meets the study criteria, they will be invited to the clinical research center to undergo examination. The screening process will last for 1 week, and will require each participant to record their symptoms in a diary without treatment during this period. Recruitment is expected to occur from August 2015 to July 2017.

### Eligibility criteria

Patients meeting the following criteria will be selected as study volunteers. The inclusion criteria for this study will include: (1) men and women aged 18 to 60 years, (2) patients with neck pain as the main symptom for at least 3 months, (3) episodes of pain lasting for over 30 min, and frequency of pain occurring at least once per month, (4) a score of 3 or more on the Visual Analogue Scale (VAS) for perceived pain intensity, (5) no acupuncture therapy within 3 months prior to study recruitment and (6) willing to participate in the study and be randomly allocated into study groups.

### Exclusion criteria

Participants meeting one of the following criteria will be excluded: (1) history of cervical intervertebral disc herniation accompanied by nerve symptoms, neck trauma or have a surgical neck fracture, spinal disease or infectious inflammatory autoimmune diseases, congenital vertebral anomalies, or compression fractures, (2) any other conditions including active skin infection, clotting disorders, administration of an anticoagulant agent, pregnancy and seizure disorders, (3) other chronic diseases including liver or kidney disease, cerebrovascular disease, leukemia, thrombocytopenia with bleeding tendency, or severe osteoporosis, (4) a severe psychiatric or psychological disorder related to severe neurosis, dementia and unable to communicate or provide self-care and (5) having taken corticosteroids, narcotics, muscle relaxants or herbal medicines to treat neck pain or any medication considered inappropriate by the recruitment investigators.

### Randomization and allocation concealment

The patients will be divided into five groups: a traditional acupuncture group (group A), a shallow-puncture group (group B), a non-acupoint acupuncture group (group C), a non-acupoint shallow-puncture group (group D) and a sham-puncture group (group E). Before randomization, all participants will be informed that they will be allocated into one of the above groups in an equal ratio, using block randomization with a block size of 5, and 35 participants are planned for each group. Randomization will occur on the second visit to the clinical research center according to the sequence generated by PEMS (Package for Encyclopedia of Medical Statistics) 3.1 software (West China School of Public Health, Sichuan, China) and overseen by an independent statistician. Randomization information will be sealed in opaque envelopes. If participants pass the screening test, a random number will be assigned sequentially.

### Blinding

All patients will be treated in the acupuncture clinical research center and will not be informed of the type of treatment that they will receive. Outcome assessors, data managers and the statistician will be blinded in this trial. To preserve masking, only the acupuncturists will have access to treatment allocation [19, 20]. However, they must learn how to use the blinding method to communicate with participants to ensure treatment blinding. The blinding principle will be operated until the data are locked down. The questionnaires regarding blinding will be completed immediately after the 5th and 10th treatments, as well as at the end of the follow-up. The success of these blinding strategies will be appraised at the end of the study by the Blinding Index (BI).

### Interventions

Participants will be randomized to the five treatment groups and receive ten sessions of treatment over a period of 5 weeks. Acupuncture treatment will be performed by a senior acupuncturist who has held a practitioner licence for more than 10 years. The participants will be asked to rest for 15 min before treatment. A constant room temperature of 23 to 25 °C will be maintained to ensure the patients are comfortable and relaxed during treatment. Subjects will be advised to lie in the prone position on the treatment couch. Interventions will be performed twice a week from Monday to Friday. Each session will last for 20 min.

The combinations of EX-HN 15 and SI 15 will be selected for the different groups based on the academic literature [21]. EX-HN 15 is an acupoint on the neck, 2 cun (1 cun is equal to the width of the patient’s thumb knuckle stripe) above GV 14 (*Dazhui*), and 1 cun lateral to the posterior median line. SI 15 is located on the back, under the 7th cervical vertebra and 2 cun lateral to GV 14. According to TCM theory, stimulation of EX-HN 15 and SI 15 can remove meridian stasis by promoting the circulation of “Qi” and blood to relieve neck pain. After the skin is sterilized with disposable 75% alcohol swabs, a sterile needle (length: 25 mm, diameter: 0.30 mm; Huatuo, Suzhou Medical Supply Factory Co., Ltd., Suzhou, China) will be inserted. The needle-punching depth in the acupoint will be 0.5 to 0.8 cun. The needle will be manipulated by lifting and twirling until the participant feels a sensation (denominated *de-qi*).

The needle in the non-acupoint will be inserted without the use of acupuncture techniques during the experiments. Non-acupoints 1 and 2 are located 1 cm lateral to EX-HN 15 and SI 15. The shallow puncture is just enough to maintain the needles in a fixed position and not enough to produce *de-qi*. For subjects receiving sham puncture, “Streitberger” needles (Asiamed Inc., Bridport, UK) with blunt tips will be used in the same areas as those in the treatment group, but will not pierce the skin. The needles will then be fixed and retained for 20 min. The treatment details in each group are illustrated in Table 1.

**Table 1:** Treatment details of each group

Group	Acupoint	Manipulation
Group A traditional acupuncture	EX-HN 15 (*Jingbailao*)	The needle-punching depth in the each acupoint is 0.5 to 0.8 cun. The sensation (denominated *de-qi*) is necessary
	SI 15 (*Jianzhongshu*)	
Group B acupoints shallow puncture	EX-HN 15 (*Jingbailao*)	The needles are pierced through the skin for 0.2 cun and not enough to produce *de-qi*
	SI 15 (*Jianzhongshu*)	
Group C non-acupoint acupuncture	Non-acupoint 1	The needle-punching depth in the acupoints is 0.5 to 0.8 cun. The sensation (denominated *de-qi*) is necessary
	Non-acupoint 2	
Group D non-acupoint shallow puncture	Non-acupoint 1	The needles are pierced through the skin for 0.2 cun and not enough to produce *de-qi*
	Non-acupoint 2	
Group E sham puncture	EX-HN 15 (*Jingbailao*)	The placebo needle tip is pressed and fixed on the skin of the acupoint
	SI 15 (*Jianzhongshu*)	

### Outcome measurements

#### Primary outcome measurement

##### Neck Pain Scale

The Northwick Park Neck Pain Questionnaire (NPQ) has been widely used in research and has been shown to have high validity and reliability for neck pain measurement [22]. It is comprised of nine items including the degree and duration of pain, symptoms including numbness, sleep and social activities, and quality of life aspects. The highest score is 100 and high scores indicate severe damage due to neck pain. The NPQ will be completed after each treatment and in the follow-up period.

#### Secondary outcome measurement

##### Pain threshold

A patient’s pain threshold (PT) is a semi-objective outcome and will be measured twice after each treatment. The PT measuring device (EP601C, East China Normal University of Science and Technology, Guangxi, China) has a 5-mA range. The positive electrode (anode) of the device is placed in the area of pain, and the other electrode is placed on the leg. According to the staircase method, the stimulus selection procedure with an increasing sequence of current stimulation from 0 mA in 2-mA steps will be presented to the patient. The patient will control the switch and press the stop key once they feel pain. The current strength (mA) on the screen will be recorded and the sequence direction will then be reversed. The descending sequence will be stopped when the participant feels pain, and the sequence points will be reversed again [23]. Three measurements will be recorded and the average PT calculated for each patient. PT will be assessed before and after treatment and the mean value calculated after the 5th and 10th treatments.

##### Short Form McGill Pain Questionnaire-2 (SF-MPQ-2)

This questionnaire is comprised of four parts including the Continuous (tender, cramping pain, aching pain, gnawing pain, heavy pain), Intermittent (splitting pain, shooting pain, sharp pain, stabbing pain, electric-shock pain, piercing), Neuropathic (hot-burning pain, cold-freezing pain, “pins and needles” or tingling, pain caused by light touch, itching and numbness) and Affective (tiring-exhausting, sickening, fearful, punishing-cruel) subscales [24]. The different aspects of pain evaluation and each item will be based on pain during the previous week. The scores are calculated by adding four different scores. High scores indicate worse neck pain.

##### Short-form 36-Item Health Survey (SF-36)

Health status will be evaluated using the Chinese version of the Medical Outcome Study Short-form 36-Item Health Survey (SF-36). The SF-36 will be used to test the correlation between health-related quality of life and related factors (sex, age, physical function and daily functioning) rated on a 5-point scale [25]. This survey will be performed before treatment, after the 5th and 10th treatments and at the end of the follow-up period.

##### Pain diary

The patients will be provided with neck pain diaries to report working time, attack times and duration of pain, accompanying symptoms and dosages of rescue medication. The patients will be instructed on how to use the diary to record information during treatment. Pain will be measured during the baseline period, after the 5th and 10th treatments and at the end of the follow-up period.

#### Follow-up

The researcher will contact the patients via telephone and a short messaging service. The follow-up period will be 3 months and monthly information will be collected. If participants discontinue the intervention protocols, the latest outcome data including changes in symptoms and medication compliance will be presented. The reasons for treatment failure will be recorded and analyzed.

#### Patient safety

Patient safety will be assessed before and after treatment to avoid adverse events (AEs) including local pain, bleeding, needle breakage, palpitations or dizziness. All unpremeditated attacks and unexpected effects will be recorded on the AE Report Form. The relationship between acupuncture treatment and AEs will be rated from 1 to 6 (1 = definitely related, 2 = probably related, 3 = possibly related, 4 = probably not related, 5 = definitely not related and 6 = unknown). A primary investigation and follow-up monitoring will be performed if AEs are reported.

#### Data monitoring and quality control

Information on demographics, neck pain duration, symptoms, curative effects and patient dropout will be recorded in the Case Report Form (CRF) at each visit. If patients quit the trial at any time, the reason will be clarified in the CRF and the dropout rate will be analyzed statistically. The files of all clinical cases will be monitored by the directors. Data will be collated and checked by two data managers. In addition, all patient data will be confidential.

In order to maintain trial quality, all researchers will receive professional training on the trial method, study technique and the method used for regular monitoring. The staff will be tested after training to ensure consistency of methods. Outcome assessments will be blinded to evaluate the recorded information during the study period. Any modifications and corrections on compliance should be discussed in compliance with standard operation procedures and recorded. The monitoring information will be regularly submitted to the directors.

#### Sample size calculation

Following an academic literature review, a pilot study on acupuncture and its analgesic effect in CNP was identified [26]. The NPQ will be used as the primary outcome measure in assessment of the analgesic effect of acupuncture in this trial. Results from the pilot study with a two-arm design showed that the mean changes in NPQ were 20.71 ± 11.91 (acupuncture group) and 24.04 ± 11.83 (control group) [26]. However, our trial is a five-arm parallel-group study with the aim of assessing the efficacy of acupuncture. The mean ± standard deviation (SD) values reported in the pilot study will be used as the expected values in the treatment group and four control groups in the present study. PEMS 3.1 software was used to perform the sample size calculation. Thus, we calculated the appropriate trial sample size with 90% power, and the significance level was 0.05. The results showed that a clinically important difference was detected using a minimum sample size of 30 individuals in each group. With a maximum allowable dropout rate of 15%, a total of 175 subjects (35 per group) are being recruited.

#### Statistical analysis

A statistical analysis will be performed using the Statistical Package for Social Sciences (SPSS, version 16.0, SPSS Inc., Chicago, IL, USA) in the Center of Acupuncture Clinical Research. A full analysis set (FAS) will be performed as the main analysis. According to the principle of intention-to-treat (ITT), any participants who randomly receive at least one treatment should be included in the main analysis dataset. The per-protocol (PP) set as the subset of FAS will include subjects who complete the study and have main variable baseline values in this trial. To be included in the safety set, patients will receive at least one safety evaluation after treatment. Statistical analysis of the main parameters will be carried out at baseline, at each visit and in the follow-up period. Frequency tables and charts will be used to analyze completed AE questionnaires.

Descriptive statistics will be performed to compare baseline information, patient characteristics and an evaluation of the credibility of the five groups. To determine the primary and secondary outcomes, the mean differences from baseline to the end of follow-up period will be compared and analyzed. Group differences for continuous variables will be analyzed using analysis of variance (ANOVA) to compare the subjective and semi-objective outcomes. The Wilcoxon rank sum test will be used to compare the outcome measurements before and after treatment. The differences in categorical variables between groups and adverse effects will be analyzed using the chi-square test. A *P* value of 0.05 or less will be considered significant.

#### Missing data and sensitivity analysis

In order to obtain the complete dataset, the researchers will receive professional training to avoid missing data. Experienced investigators will contact participants through messaging and telephone calls at fixed treatment periods. In addition, the participants will receive economic compensation after the trial. For missing data, we will analyze the underlying reason and use an imputation adjustment approach. The method of last observation carried forward (LOCF) will be carried out for imputation of missing values. After the main analysis, sensitivity analysis of the various datasets will be used to evaluate the impact of missing data on the trial results.

#### Ethical considerations

This research was designed in accordance with the principles of the Declaration of Helsinki. The trial protocol has been approved by the Ethics Committee of Guangdong Provincial Hospital of Chinese Medicine (Reference number B2015-058-01), and is registered on the primary registry in the World Health Organization (WHO) registry network (Chinese Clinical Trial Registry: No. ChiCTR-IOR-15006886). Signed consent will be obtained from each patient after they are informed of the procedures, possible risks of the trial and their right to discontinue participation. In addition, for those who develop several AEs, the Ethics Committee will be informed and medical treatment will be provided until the problem is resolved.

#### Monitoring and publications

An independent board from the Center of Acupuncture Clinical Research will monitor progression and provide advice when necessary. The Data Monitoring Committee has been established independently in the hospital (Traditional Chinese Medicine Hospital of Guangdong Province) and has claimed that there is no conflict of interest.

This RCT protocol was developed following the Standard Protocol Items: Recommendations for Interventional Trials (SPIRIT) Checklist (see Additional file 1) [27]. We plan to publish the protocol and results according to the guidelines of Consolidated Standards of Reporting Trials (CONSORT) and Revised Standards for Reporting Interventions in Clinical Trials of Acupuncture (STRICTA) [28, 29]. All staff who participated in the study will be included in the authorship and in the “Competing interests” statement.

## Discussion

Acupuncture has been practiced for more than two millennia in China. In the practice of traditional acupuncture analgesia, it is generally acknowledged that structures and physiological functions are modulated by 14 energy meridians and the hypothetical “Qi” is usually translated as energy [30]. Acupuncturists with different experiences might have the different methods to operate *de-qi* (bring about the desired sensation) for the acupuncture intervention. Findings from clinical research strongly suggested that these specific differences of interventions have a major impact on the response to the curative effect [31]. Therefore, further trials are required to investigate the differences in these methods.

According to TCM theory, acupoints can be selected in three ways: an acupoint can be selected at a local lesion (with the pain area as acupoint *Shu*), far away from the local lesions, such as on limbs, and in accordance with symptoms. For non-acupoints at local lesions, the possible mechanisms of the analgesic effect may include the gate control theory triggered by neural pathways, alterations in local circulation and the immune environment [32]. Moreover, for shallow needle insertion, some researchers believe that shallow puncture can stimulate afferent nerves and activate the corresponding brain regions to produce a “limbic touch response” [33, 34]. Previous studies have also suggested that non-acupoints beyond the meridian and shallow puncture have an analgesic effect in subjects with fibromyalgia and other chronic pain conditions [35, 36]. Although the use of the “Streitberger” needle with a blunt tip has been supported in studies and reviews, it was reported that nearly half of the subjects could distinguish it from the real treatment in pain disorders [14, 37]. Therefore, further trials are required to investigate these differences. It is necessary to establish a standard for the control (sham) in clinical research due to the unknown analgesia mechanisms of traditional acupuncture.

Clinical trials of traditional acupuncture for chronic pain, with optimal control groups, are rare, with the exception of the use of standard outcome measures and rigorous blinding of participants to treatment assignment [38]. However, few guidelines exist for conducting appropriate sham acupuncture, for the depth of the inserted needle or the parameter for needle stimulus [39, 40]. This randomized placebo-controlled trial addresses the urgent need to provide evidence-based control groups for adults with CNP and to determine the evidence for the specificity of acupoints. At the end of this clinical trial, we expect that the findings will provide evidence to answer the question: is acupuncture more effective than a placebo?

### Trial status

The participants are beginning to be recruited for this study. The trial is designed to be completed by 31 December 2017.

## Additional file

Additional file 1: SPIRIT Checklist. (DOC 146 kb)

## Abbreviations

AEs: Adverse events
ANOVA: Analysis of variance
BI: Blinding index
CNP: Chronic neck pain
CONSORT: Consolidated Standards of Reporting Trials
CRF: Case Report Form
FAS: Full analysis set
ITT: Intention-to-treat
LOCF: Last observation carried forward
NPQ: Northwick Park Neck Pain Questionnaire
PP: Per-protocol
PT: pain threshold
SD: Standard deviation
SF-36: Short-form 36-Item Health Survey
SF-MPQ-2: Short Form McGill Pain Questionnaire-2
STRICTA: Revised Standards for Reporting Interventions in Clinical Trials of Acupuncture
TCM: Traditional Chinese medicine
VAS: Visual Analogue Scale
